# The Relationship Between Work-Disability Duration and Claimant’s Expected Time to Return to Work as Recorded by Workers’ Compensation Claims Managers

**DOI:** 10.1007/s10926-016-9656-z

**Published:** 2016-07-26

**Authors:** Amanda E. Young, Elyssa Besen, Joanna Willetts

**Affiliations:** Center for Disability Research, Liberty Mutual Research Institute for Safety, 71 Frankland Road, Hopkinton, MA 01748 USA

**Keywords:** Recovery expectations, Disability management, Work-disability prevention, Workers’ compensation, Return-to-work, Prognostic factors

## Abstract

*Purpose* This research sought to determine whether there is a relationship between claimants’ expected time to return to work (RTW) as recorded by claims managers and compensated days of work disability. *Methods* We utilized workers’ compensation data from a large, United States-based insurance company. RTW expectations were collected within 30 days of the claim being reported and these were compared with the termination of total temporary indemnity payments. Bivariate and hierarchical regression analyses were conducted. *Results* A significant relationship between expected time to RTW and compensated disability duration was observed. The unadjusted correlation between work-disability duration and expected time to RTW was .25 (*p* < .001). Our multivariate model explained 29.8 % of the variance, with expected time to RTW explaining an additional 9.5 % of the variance in work-disability duration beyond what was explained by the covariates. *Conclusion* The current study’s findings support the hypothesis that claimant RTW estimates as recorded by claims managers are significantly related to compensated-disability duration, and the relationship is maintained after controlling for variance that can be explained by other variables available within workers’ compensation databases.

## Introduction

With an increasing number of studies finding that a worker’s medical condition incompletely explains return to work (RTW) following occupational injury, the role of psychosocial influences has come into question [1, 2]. Of the psychosocial variables that have been examined, “RTW expectation” has frequently been found to relate to outcomes [3, 4]. Questions are now being raised regarding whether a worker’s expectations for RTW can be used in a clinical setting to gain an understanding of likely future outcome.

The relationship between RTW expectations and RTW outcomes has been observed not only in numerous geographical and social settings, but also across a variety of health conditions, disability durations, and methods of scientific inquiry [5]. However, while study results have indicated a relationship between expectations and RTW, our understanding of the relationship is still limited. The reasons for this are numerous, with some of the most important being: inconsistency in findings, study sample sizes that have limited the inclusion of covariates, and data being collected by researchers rather than those involved in the claims process.

Regarding inconsistency of findings, research has indicated that a claimant’s self-assessment may be more or less accurate depending on their condition. For example, in their analysis of 1040 workers’ compensation claimants, Gross and Battié found that recovery expectations predicted recovery in workers filing injury claims for back pain, but not other musculoskeletal conditions including sprains, strains, or pain of other body parts besides the back, other injuries such as fracture, dislocation, or amputation, nor other compensable conditions such as carpal tunnel [6].

Regarding sample size, studies have tended to have sample sizes that have restricted their analytical options and the number of covariates that could be included. Of the studies that have investigated the relationship between expectations and RTW outcomes, the maximum sample size was 1566 [7]. This study was conducted in Canada and found that four measures of recovery explained one-sixth of the variation in time receiving benefit. While expectations regarding RTW were not found to be individually predictive of time receiving benefits, this is likely due to the question about whether or not the respondent thought they would recover enough to return to their usual job. The next largest sample comprised 1068 people with a workers’ compensation claim for back pain in Washington State [8]. In this study, they found that very low recovery expectations (operationalized as being very uncertain about whether or not they would be working in 6 months’ time) were significant independent predictors of chronic work disability. Four other studies have had samples sizes of approaching 1000 [6, 9–11]. In the majority of the remainder of studies, sample size has tended to be around 500 [e.g., 12–17].

Regarding the impact of those collecting the data, to date, data has mainly been collected by research staff. It is possible that claimant responses are influenced by *who* is asking the question. For example, workers may be more willing to give an honest response to an un-invested party. Or, they may give what they believe to be a socially desirable (biased) response to someone with an interest in their specific case. While there are some studies in which those collecting the data have been directly involved in the RTW process [e.g., 18–20] in these cases data has been collected by a treating health care professional: in the study by Waylett-Rendall and Niemeyer [20], data were collected at the point of care by therapists working in the hand therapy program; in the case of Nieuwenhuijsen et al. [18], data were collected by an occupational physician; and in the study by Gross and Battié [19], data were abstracted from the treating facility’s database. As yet, there has been no study of the relationship between RTW expectations data as collected by an insurer representative as part of the case management process and work-disability duration. Given that this stakeholder group is one of the most likely to implement interventions based on the information received, understanding the impact of who is asking is important for both research and clinical reasons.

### Aims and Hypotheses

The research sought to determine whether there is a relationship between claimants’ expected time to return to work as recorded by claims managers in the administrative database of a large workers’ compensation insurer and compensated days of work disability. We hypothesized that claimant estimates would be related to work-disability durations as calculated using payments for missed work time. In addition, we sought to determine if variance in work-disability duration that is accounted for by claimants’ RTW expectations is greater than can be achieved with demographic and injury variables contained within workers’ compensation (WC) administrative databases.

## Methods

In the current study, we utilized the WC data from a large, United States-based insurance company. The data covers claims from a variety of organizations with different workforce sizes and from various industries. We focused on data pertaining to claimants aged 18–80 who had at least 7 days of compensated temporary total disability (TTD) and who reported expecting to return to work. The reason for focusing on people with at least 7 days of TTD was that this timeframe represents a substantial period of time away from the workplace. As such, findings are likely to be more applicable to the subset of injured workers that has the potential to benefit most from work-disability prevention initiatives. So as to allow for at least 18 months of claims maturation, data were extracted for persons with an accepted claim that occurred from January 1, 2010 until December 31, 2013.

As part of the insurer’s claims management process, claimants who are off work, but not necessarily receiving indemnity payments, were asked about their RTW expectations. For the current study, we restricted our sample to claimants who provided an estimated RTW date within 30 days of the date that their injury was first reported to the workers’ compensation insurer. The reason for this is that our focus is on early risk prediction. We also restricted the sample to claims that had just one episode of TTD in the 365 days following RTW expectation data collection. Our justification for this is that while it is likely that the majority of respondents would reference their most immediate RTW when asked about their expectations, we could not be sure of this. Excluding people with multiple episodes of work-disability (TTD) means that we could be confident of the RTW the claimant was referencing. Similarly, in instances where a claimant had multiple claims within our data collection period (2010–2013), all claims for this individual were also excluded so as to avoid confusion about what incident a person was referencing when making RTW expectations.

**Fig. 1:**
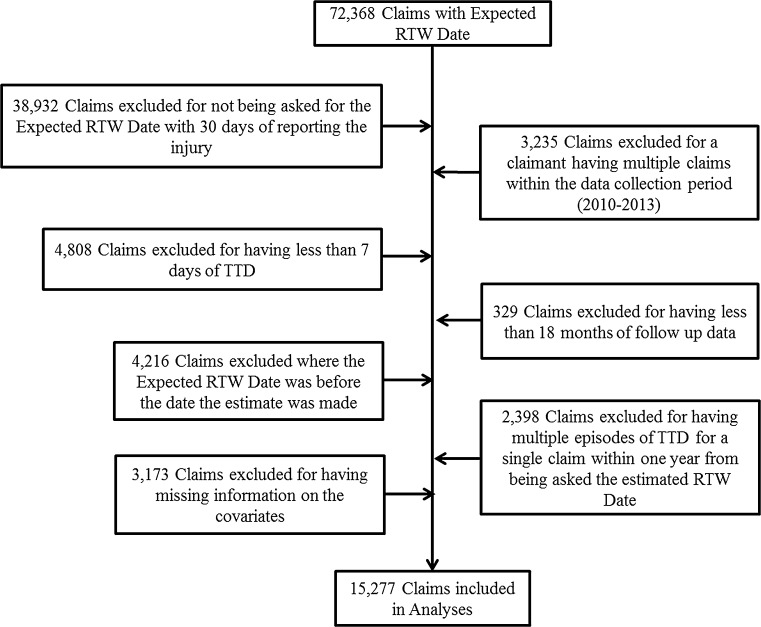
Flow chart illustrating sample lost due to exclusion criteria

Consistent with prior studies of disability recurrence, we considered a new episode of TTD to have occurred if there was more than a 7-day gap between TTD payments [21]. If the duration between TTD payments was 7 days or less, it was considered a single episode. We also excluded claimants who received a lump sum payment within 365 days of the date that the prediction was made of when a claimant would return to work. The reason for this is that in such cases, the end of TTD payments is a less reliable proxy for RTW. Claimants with missing data were also excluded. After the sample restrictions, 15,277 claims were included in our analyses. Figure 1 illustrates where sample was lost due to the various restrictions.

**Fig. 2:**
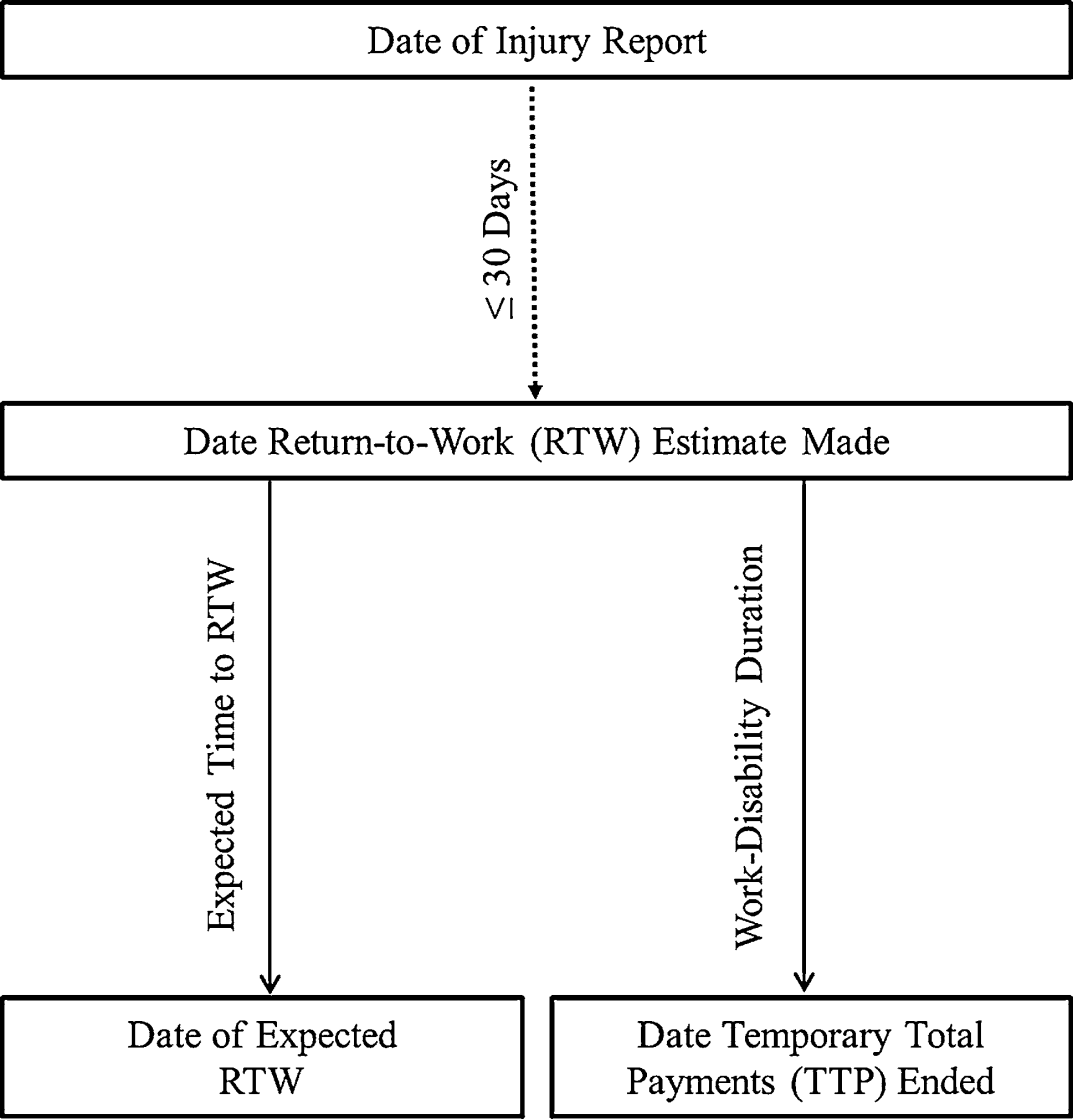
Time points used to calculate expected time to return to work and work-disability duration

Three key dates were utilized in this study. These were the date of injury report, the date the RTW estimate was made, and the date that consecutive temporary total disability (TTD) payments ended (note that gaps of 7 days or less were ignored). The date that the injury was reported was used to assess whether an estimate for expected time to RTW was made within 30 days of the injury report day. The date the RTW estimate was made was then used to calculate (1) the expected length of time (days) to RTW and (2) the actual length of time (days) to RTW, estimated using the date that TTD payments ended. The key dates and associated measures are illustrated in Fig. 2.

### Measures

#### Outcome Variable

Work-disability duration was the outcome variable for our analyses. It was calculated as the number of days from the date at which claimants first made an estimate of when they would RTW until the date at which consecutive TTD payments finished. TTD was considered to have ended when no TTD days were taken for at least a 7-day consecutive period. Ignoring gaps in payment is consistent with prior research [22–25]. We opted to use a relatively conservative ignored-gap period as we felt that extending the period beyond 7 days would result in defining people who had attempted to RTW, but were unable to maintain their rehabilitation gains (i.e. experienced a work-disability recurrence [21]) as experiencing a single episode of work disability. In cases of work-disability recurrence, we would not expect that a claimant’s original RTW estimates would relate to the end of a later episode of work disability. Work-disability duration was top coded at 365 days in cases where disability duration exceeded 1 year. This occurred in 1010 claims. In the analyses, the natural log of disability duration was used to address issues with normality.

#### Predictor Variable

The main predictor variable for our analyses was the claimant’s expected time to RTW. A recent review of research into RTW expectations found much diversity in the way expectations have been assessed [5]. Based on the review, the following measure was offered as a means of advancing the field: (1) “Do you expect to go back to work?”, Response options: “yes”, “no”, “unsure”. If “yes” or” unsure”: (2) “If you had to estimate your time-frame for going back to work, what would it be? Response options: “Time from today: _____days/weeks/months (circle one)”. While the exact wording used by the claims managers to collect the RTW expectations data is not standardized, the data collected is consistent with what was recommended based on the review [5].

For this study expected time to RTW was calculated for people who responded that they did expect to RTW, or that they were unsure but were able to give an approximate timeframe for doing so. The expected time to RTW was defined as the number of days from the date the claimant was first asked to estimate when he/she would RTW, until the date the claimant reported expecting to RTW. The expected length of time until RTW was top coded at 365 days in cases where the length of time exceeded 1 year. This occurred in 56 claims. The natural log of the expected length of time until returning to work was used in analyses to address issues with normality.

#### Covariates

The following 11 covariates were used: age, tenure, gender, industry, surgery post-RTW expectations collection, comorbidity, perceived pain, prior injury, diagnosis, jurisdiction, and number of days between the report date and the date the RTW expectation was made.

- Age was measured in years based on the claimant’s age at the time of injury.
- Tenure was also measured in years based on a claimant’s organizational tenure at the time of injury. Tenure was top coded at 25 years (occurred in 643 cases); in analyses, the natural log of the length of tenure was used to address issues with normality.
- Gender was coded 1 for female and 0 for male.
- Ten industry groupings, which roughly correspond to the U.S. Department of Labor’s Standard Industry Classification (SIC) groups [26], were included in the analyses: agriculture, forestry and fishing, construction, finance and insurance, manufacturing, mining, retail trade, services, transportation, public administration, and wholesale trade.
- A surgery indicator was used in analyses to control for claimants receiving surgery after the claimant had made a prediction about the date at which he/she would RTW. This indicator was constructed based on two criteria from reviewing the claimant’s medical bills for the first year following the date the injury occurred. First, the claimant needed to have a bill containing at least one Current Procedural Terminology Code (CPT) in the broad category of surgery (ranging from 10,000 to 69,990) within the first year following the date at which the RTW estimate was made. Second, the claimant also needed to have a bill containing at least one CPT code in the broad category of Anesthesia (ranging from 00100 to 01999) occurring within 6 days of the CPT surgery code to allow for minor administrative billing inconsistencies. Claimants were coded 1 for having a surgery if both criteria were met and 0 if they did not meet both criteria after making a prediction about the RTW date.
- Comorbidity was coded 1 if the claimant reported having hypertension and/or diabetes and 0 if the claimant had neither condition. These data were collected by the claims manager as self-reported by the claimant.
- Perceived pain level was coded on an eleven-point scale from 0 indicating “no pain” to 10 indicating “lots of pain” [27]. These data were collected by the claims manager as self-reported by the claimant.
- Prior injury was categorized into: claimant did not have a prior injury (reference category), claimant had a prior injury related to the current claim, and claimant had a prior injury that was unrelated to the current claim. These data were collected by the claims manager as self-reported by the claimant.
- The primary diagnosis which best captured the reason for the claim (as defined by the claims manager and based on the self-report of the claimant) was assigned using the International Classification of Diseases, ninth revised edition (ICD-9). The diagnoses were collapsed into fourteen different groups (see Table 1 for full listing of diagnosis groups). An analysis was conducted whereby the codes applied by the case manager were compared with medical billing information. In 82.14 % of cases there was at least one bill coded to the diagnosis groupings displayed in Table 1. In 91.62 % of cases there was concordance at the ICD-9 chapter level.

**Table 1:** Descriptive statistics and hierarchical analyses results for administrative variables and expected time to RTW predicting work-disability duration (N = 15,277)

Predictors	Mean (SD)	Median (IQR)	Model 1: Administrative variables			Model 2: Administrative variables + Expected time to RTW		
			β	Coef (S.E.)	95 % CI			
						β	Coef (S.E.)	95 % CI
Expected time to RTW	33.4 (42.8)	21 (7–42)				.346***	.610 (.013)	.583:.636
Age	42.6 (13.1)	43 (32–53)	.053***	.009 (.001)	.006:.011	.039***	.006 (.001)	.004:.009
Tenure	5.8 (7.0)	2.6 (.6–8.4)	−.005	−.006 (.010)	−.025:.013	−.010	−.012 (.009)	−.030:.005
Pain	5.4 (2.3)	5 (4–7)	.158***	.142 (.007)	.129:.156	.112***	.101 (.006)	.089:.114
Days from report to expectation estimate	9.7 (7.0)	9 (4–13)	−.026***	−.008 (.002)	−.012:−.003	−.057***	−.017 (.002)	−.021:−.013

	f (%)							
Female^a^	4924 (32.2 %)		.002	.010 (.035)	−.059:.079	.012	.052 (.033)	−.013:.117
Comorbidity (yes)^b^	3310 (21.7 %)		.017*	.088 (.040)	.010:.165	.017*	.085 (.037)	.012:.157
Prior injury related to claim (yes)^c^	1694 (11.1 %)		.023**	.151 (.050)	.052:.250	.021**	.143 (.047)	.050:.236
Prior injury unrelated to claim (yes)^c^	2781 (18.2 %)		.011	.059 (.041)	−.021:.138	.011	.058 (.038)	−.017:.133
Surgery (yes)	2877 (18.8 %)		.291***	1.560 (.043)	1.476:1.643	.243***	1.303 (.040)	1.224:1.382
Industry								
Services^d^	4463 (29.2 %)							
Agriculture, forestry & fishing	110 (.7 %)		.001	.028 (.183)	−.331:.387	.000	.001 (.172)	−.336:.338
Mining	269 (1.8 %)		.033***	.525 (.125)	.281:.770	.022**	.347 (.117)	.117:.576
Construction	758 (5.0 %)		.042***	.403 (.076)	.253:.552	.025***	.237 (.072)	.097:.377
Manufacturing	1971 (12.9 %)		.013	.083 (.053)	−.021:.187	.010	.062 (.050)	−.036:.159
Wholesale trade	3123 (20.4 %)		.058***	.302 (.047)	.210:.394	.020*	.102 (.044)	.015:.188
Retail trade	980 (6.4 %)		.018*	.150 (.067)	.018:.282	.013	.110 (.063)	−.014:.234
Finance, insurance and real estate	2920 (19.1 %)		−.005	−.029 (.045)	−.118:.060	.006	.034 (.043)	−.049:.118
Public administration	606 (4.0 %)		−.005	−.057 (.082)	−.217:.104	−.001	−.011 (.077)	−.161:.140
Transport, communication, & electric	77 (.5 %)		.000	−.005 (.232)	−.459:.449	.005	.145 (.217)	−.281:.571
Diagnosis								
Sprains and strains of joints/adjacent muscles^e^	6030 (39.5 %)							
Diseases of the digestive system	634 (4.2 %)		−.017*	−.184 (.082)	−.345:−.022	−.038***	−.401 (.077)	−.553:−.250
Diseases of the nervous system	169 (1.1 %)		.033***	.658 (.148)	.368:.947	.017*	.348 (.139)	.077:.620
Contusion or crushing injury	997 (6.5 %)		−.040***	−.338 (.065)	−.465:−.212	−.024***	−.201 (.061)	−.320:−.082
Dislocation	518 (3.4 %)		.025***	.293 (.089)	.119:.466	.005	.061 (.083)	−.103:.224
Fracture of lower limb	1417 (9.3 %)		.081***	.586 (.056)	.475:.697	.016*	.117 (.054)	.011:.222
Fracture of skull, spine or truck	265 (1.7 %)		.017*	.280 (.119)	.047:.513	−.001	−.011 (.112)	−.230:.208
Fracture of upper limb	1218 (8.0 %)		.038***	.294 (.060)	.176:.412	−.006	−.046 (.057)	−.157:.065
Open wound	936 (6.1 %)		−.036***	−.316 (.068)	−.448:−.183	−.031***	−.270 (.063)	−.394:−.145
Other injury and poisoning	673 (4.4 %)		−.009	−.097 (.077)	−.248:.054	−.008	−.082 (.072)	−.224:.060
Arthropathies and related disorders	417 (2.7 %)		.033***	.422 (.096)	.233:.611	.024***	.309 (.090)	.132:.486
Dorsopathies, osteopathies, and related disorders	868 (5.7 %)		.070***	.631 (.069)	.496:.767	.059***	.538 (.065)	.410:.665
Rheumatism (excluding back)	863 (5.7 %)		.059***	.538 (.070)	.400:.676	.026***	.238 (.066)	.108:.368
Other disease	272 (1.8 %)		.006	.101 (.117)	−.128:.330	.006	.099 (.110)	−.116:.314
State								
NY^f^	1123 (7.4 %)							
AK	66 (.4 %)		.005	.160 (.238)	−.307:.627	.002	.056 (.223)	−.382:.494
AL	160 (1.1 %)		.014	.295 (.159)	−.017:.607	.003	.056 (.149)	−.237:.349
AR	103 (.7 %)		.002	.061 (.194)	−.319:.441	−.003	−.083 (.182)	−.440:.274
AZ	245 (1.6 %)		.010	.174 (.133)	−.086:.434	.012	.199 (.125)	−.045:.443
CA	1882 (12.3 %)		.065***	.416 (.071)	.276:.555	.049***	.315 (.067)	.183:.446
CO	188 (1.2 %)		.016*	.298 (.149)	.006:.590	.007	.134 (.140)	−.140:.408
CT	208 (1.4 %)		−.008	−.143 (.142)	−.421:.136	−.009	−.159 (.133)	−.420:.103
DC	28 (.2 %)		−.020**	−.969 (.359)	−1.673:−.264	−.016*	−.762 (.337)	−1.424:−.101
DE	91 (.6 %)		.009	.244 (.205)	−.157:.646	.011	.298 (.192)	−.079:.675
FL	333 (2.2 %)		−.028***	−.395 (.118)	−.626:−.164	−.029***	−.423 (.111)	−.640:−.206
GA	327 (2.1 %)		.039***	.563 (.119)	.329:.796	.024**	.341 (.112)	.122:.560
HI	107 (.7 %)		.011	.271 (.190)	−.102:.645	.022**	.560 (.179)	.209:.910
IA	204 (1.3 %)		−.005	−.087 (.144)	−.369:.196	−.003	−.064 (.135)	−.329:.202
ID	59 (.4 %)		−.012	−.416 (.251)	−.908:.077	−.006	−.186 (.236)	−.648:.276
IL	1037 (6.8 %)		.021*	.178 (.082)	.018:.339	.024*	.196 (.077)	.046:.347
IN	270 (1.8 %)		−.012	−.190 (.128)	−.441:.061	−.018*	−.293 (.120)	−.528:−.058
KS	167 (1.1 %)		−.005	−.091 (.157)	−.399:.217	−.003	−.057 (.147)	−.346:.232
KY	302 (2.0 %)		.018*	.267 (.123)	.027:.508	.012	.181 (.115)	−.045:.407
LA	145 (1.0 %)		.035***	.764 (.166)	.437:1.090	.023***	.501 (.156)	.195:.807
MA	661 (4.3 %)		.004	.045 (.092)	−.136:.226	.010	.101 (.087)	−.069:.271
MD	366 (2.4 %)		−.003	−.038 (.113)	−.260:.185	.004	.058 (.106)	−.151:.266
ME	49 (.3 %)		.001	.049 (.274)	−.489:.586	.003	.112 (.257)	−.392:.617
MI	546 (3.6 %)		.010	.109 (.099)	−.084:.303	.000	−.003 (.093)	−.185:.179
MN	257 (1.7 %)		−.021**	−.341 (.131)	−.597:−.084	−.007	−.115 (.123)	−.357:.126
MO	287 (1.9 %)		−.029***	−.452 (.125)	−.697:−.207	−.019*	−.291 (.117)	−.521:−.061
MS	204 (1.3 %)		.020**	.369 (.143)	.088:.650	.009	.157 (.135)	−.107:.421
MT	57 (.4 %)		.015*	.527 (.255)	.027:1.027	.016*	.547 (.239)	.078:1.016
NC	264 (1.7 %)		.061***	.982 (.129)	.729:1.234	.047***	.750 (.121)	.513:.988
NE	89 (.6 %)		−.012	−.339 (.208)	−.746:.069	−.009	−.238 (.195)	−.620:.144
NH	162 (1.1 %)		−.004	−.082 (.167)	−.409:.245	−.003	−.053 (.156)	−.360:.254
NJ	871 (5.7 %)		−.043***	−.393 (.085)	−.560:−.226	−.037***	−.336 (.080)	−.493:−.179
NM	94 (.6 %)		.009	.242 (.202)	−.154:.638	−.002	−.051 (.190)	−.423:.321
NV	104 (.7 %)		.019*	.491 (.193)	.113:.870	.014	.345 (.181)	−.010:.700
OH	131 (.9 %)		.024**	.547 (.174)	.206:.888	.033***	.752 (.163)	.432:1.072
OK	190 (1.2 %)		.025***	.481 (.149)	.188:.773	.014	.258 (.140)	−.017:.533
OR	231 (1.5 %)		.018*	.303 (.137)	.035:.571	.014	.238 (.128)	−.014:.489
PA	844 (5.5 %)		.028**	.255 (.086)	.086:.425	.020*	.186 (.081)	.027:.345
RI	5 (.0 %)		.006	.639 (.842)	−1.011:2.288	.004	.494 (.790)	−1.054:2.042
SC	136 (.9 %)		−.003	−.065 (.171)	−.399:.270	−.005	−.110 (.160)	−.424:.204
SD	28 (.2 %)		−.007	−.361 (.360)	−1.066:.343	−.012	−.598 (.337)	−1.260:.063
TN	419 (2.7 %)		−.010	−.124 (.108)	−.336:.088	−.012	−.156 (.102)	−.355:.043
TX	813 (5.3 %)		.057***	.536 (.089)	.362:.710	.023**	.214 (.084)	.050:.378
UT	94 (.6 %)		.012	.309 (.202)	−.087:.705	.012	.315 (.189)	−.057:.686
VA	506 (3.3 %)		.017	.194 (.101)	−.004:.392	.017*	.199 (.095)	.013:.385
VT	81 (.5 %)		−.010	−.287 (.217)	−.712:.138	−.006	−.171 (.203)	−.569:.228
WA	63 (.4 %)		−.001	−.029 (.244)	−.507:.449	.004	.117 (.229)	−.332:.566
WI	557 (3.7 %)		−.036***	−.408 (.100)	−.603:−.212	−.021*	−.240 (.094)	−.423:−.056
WV	123 (.8 %)		.007	.168 (.179)	−.183:.518	.011	.252 (.168)	−.077:.581
Intercept				2.615 (.069)	2.481:2.750		2.842 (.064)	2.716:2.969
R^2^				.202***				.298***
Change in R^2^								.095***

*IQR* interquartile range 25–75 %

* *P* < .05; ** *P* < .01; *** *P* < .001

^a^Male is the reference group

^b^Does not have hypertension or diabetes is the reference group

^c^Has no prior injury is the reference group

^d^Services is the reference group

^e^Sprains and strains of joints and adjacent muscles is the reference group

^f^NY is the reference group
- Jurisdiction was coded based on the state in which the injury occurred.
- The number of days between the injury report date and the date the RTW expectation was made was measured continuously in days.

### Analyses

Given that only a small proportion of cases were top coded for work-disability duration (6.6 %), hierarchical regression was used to estimate the relationship between claimant’s expected time to RTW and work-disability duration, as well as to assess whether RTW expectations account for additional variance in work-disability duration beyond that which could be accounted for by selected covariates contained within the WC insurer’s administrative databases. In the first step of the analyses, the covariates, were added to the model predicting work-disability duration. In the second step, the expected time to RTW was added to the model. The change in the amount of variance accounted for (R^2^) in the first model compared to the second model was used to examine the amount of additional variance accounted for by RTW expectations. To test the hypothesis that claimants’ estimates for expected RTW are related to actual work-disability duration, the coefficient for the expected time to RTW in the second model was used. All analyses were conducted using STATA 13.1 (College Station, TX). The user-written program “hireg” was utilized to implement the hierarchical regression model [28].

## Results

In total, 15,277 claims were included in the analyses. The mean work-disability duration was 78.7 days (SD 102.8), and the mean expected time to return to work was 33.4 days (SD 42.8). Slightly more than two-thirds (67.8 %) of the claims were for men. Claimants’ ages ranged from 18 to 80 years with an average age of 42.6 years and the average length of tenure was 5.8 years. Approximately a fifth (21.7 %) of participants reported having a comorbid health condition (having hypertension and/or diabetes). The average perceived pain level was 5.4. The majority of claimants did not have a prior injury (70.7 %), while 11.1 % of claimants had a prior injury that was related to their current claim and 18.2 % had a prior injury that was not related to their current claim. The average number of days between the report date for the claim and when a claimant made a prediction about the date at which he/she would RTW was 9.7 days. Additional descriptive statistics can be found in Table 1.

Although the average expected time to RTW was less than the time it took for TTD payments to cease (33 vs. 79 days), analysis revealed a significant relationship between expected time to RTW and work-disability duration. The unadjusted correlation between work-disability duration and the expected time to RTW was .25 (*p* < .001). To examine how closely work-disability duration aligned with the expected time to RTW, we categorized both variables into eight categories and conducted cross-tabulations among the categories (see Table 2). In line with our expectations, within each of the length-of-disability categories, the largest percentage of claims were in the same or similar number-of-days category for the expected time to RTW. The most accurate group were those who expected to be back at work in 1-7 days, with 43 % of this group making a correct estimation. Approximately 10 % of the sample had an expected time to RTW of 7 days or less and a work-disability duration of 7 days or less. In contrast, less than 1 % of the sample had an expected time to RTW of 181 days or longer and a work-disability duration of 7 days or less. Those who expected to return to work in more than 7 days tended to be less accurate, but in most cases the rate of accuracy was still greater than 20 %. The rate of accuracy for those who expected to be off work for more than 7 days was 22 %. Overall, 28 % of the sample made estimates that were within 7 days (±) of their TTD payments ending; and 41 % were within 14 days (±) of when their TTD payments ended.

**Table 2:** Percentage and number of claims in work-disability duration categories by expected time to return to work (RTW) categories

Expected time to RTW (days)	Work-disability duration (days)								
	1–7 (n = 2652)	8–14 (n = 1776)	15–30 (n = 2619)	31–60 (n = 2774)	61–90 (n = 1542)	91–180 (n = 1797)	181–365 (n = 1107)	>365 (n = 1010)	Total (N = 15,277)
1–7	**43.0** **%**	14.7 %	14.7 %	11.3 %	4.9 %	5.1 %	2.9 %	3.5 %	25.1 %
	**1650**	563	562	433	187	194	112	133	1650
8–14	14.4 %	**20.9** **%**	22.7 %	17.1 %	7.9 %	7.5 %	4.5 %	4.9 %	16.0 %
	351	**511**	555	418	194	183	111	120	2443
15–30	10.0 %	10.5 %	**22.8** **%**	20.6 %	10.1 %	11.9 %	7.5 %	6.6 %	22.7 %
	348	365	**791**	715	351	413	261	230	3474
31–60	6.5 %	7.6 %	14.6 %	**24.9** **%**	14.5 %	14.8 %	9.2 %	8.0 %	21.2 %
	210	247	473	**805**	468	478	299	259	3239
61–90	4.8 %	4.3 %	12.6 %	20.1 %	**15.2** **%**	21.5 %	11.1 %	10.4 %	8.0 %
	58	53	154	245	**185**	263	136	127	1221
91–180	2.0 %	3.0 %	7.9 %	16.0 %	15.6 %	**26.5** **%**	17.7 %	11.5 %	5.7 %
	17	26	68	138	135	**229**	153	99	865
181–365	6.9 %	6.9 %	6.9 %	6.9 %	11.7 %	22.1 %	**20.0** **%**	18.6 %	0.9 %
	10	10	10	10	17	32	**29**	27	145
>365	14.3 %	1.8 %	10.7 %	17.9 %	8.9 %	8.9 %	10.7 %	**27.0** **%**	0.4 %
	8	1	6	10	5	5	6	**15**	56

Bolded cells represent claims where the expected time to RTW was the same at the work-disability duration

*RTW* return to work

The adjusted relationship between expected time to RTW and work-disability duration while controlling for selected variables contained within the WC insurer’s administrative database was tested using a two-step analytical procedure. In the first step of the analyses, the covariates which represent the variables typically collected in administrative databases explained 20.2 % of the variance in work-disability duration (see Table 1). In the second step of the analyses, when the expected time to RTW was added to the model, the model then explained 29.8 % of the variance, with the expected time to RTW explaining an additional 9.5 % of the variance in work-disability duration beyond what was explained by the covariates. As hypothesized, the claimants’ expected time to RTW was related to work-disability duration, with a greater expected time to RTW being associated with an increase in work-disability duration (β = .346, *p* < .001).

In addition to the main analyses, we conducted a series of sensitivity analyses. As not all claimants were receiving indemnity payments at the time that the RTW expectation estimate was given (n = 2291), we conducted the aforementioned analyses limiting our sample to claimants who were receiving indemnity payments for TTD at the time that the RTW expectation estimate was given. In addition, to ensure that the expectation estimate was made reasonably close to the start of TTD, the sample was restricted to those claimants who made the estimate within 2 weeks of starting to receive indemnity payments. Results were consistent in the sensitivity analysis sample with the expected time to RTW being positively related to work-disability duration (β = .314, *p* < .001) and explaining an additional 7.8 % of the variance in work-disability duration beyond the covariates. The results are available upon request.

We also conducted sensitivity analyses limiting our sample to claimants who had finished receiving indemnity payments for TTD within 365 days of RTW expectation data being collected. Findings remained consistent. The correlation between expected time to RTW and TTD duration was .26 (*p* < .001), and the expected time to RTW explained an additional 10.6 % of the variance in work-disability duration after adjusting for the covariates (full model explained variance = 27.4 %). Finally, we compared the main sample, with claimants who were excluded from the sample due to missing covariate data. Claimants with missing data had significantly (p < .001) shorter lengths of disability (mean = 60 days) and expected time to RTW was sooner (mean = 30 days) than those in the main sample (work-disability duration—mean = 79 days; expected time to RTW—mean = 33 days). For the claimants who were excluded as a result of missing information, the correlation between work-disability duration and expected time to RTW was slightly higher than for the study sample (r = .28 as compared to r = .25).^1^

## Discussion

While past research has consistently found that workers’ expectations for return to work are predictive of their eventual outcome, understanding of the relationship has been limited by inconsistency in findings, small and restricted study sample sizes and the potential influence of the person collecting the data. Although estimates tended to be more optimistic than was actually the case, the current study’s findings support the hypothesis that claimant RTW estimates as recorded by claims managers are significantly related to compensated-disability duration, and that relationship is maintained after controlling for variance that can be explained by other variables contained within workers’ compensation data. With being true regardless of whether or not the claimant was in receipt of wage replacement payments at the time of being asked about their RTW expectations. Accuracy was highest for people who expected to RTW within a week. Although accuracy was found to decrease in cases where the estimated time to RTW was greater than 7 days, in more than 40 % of the sample the predicted time to RTW was within 14 days of the end of their TTD payments.

Study findings are consistent with a relationship observed in a recent investigation into RTW following carpal tunnel release [29] where it was found that expected time to RTW explains 18 % of the variance when compared to time to a full RTW. This is despite the current sample’s estimated time to RTW being longer than was the case in the earlier study (33.4 days vs. 18.9 days) and our larger sample size (N = 15,277 vs. N = 65). Such findings add further support to the idea that injured worker’s expectations for RTW can be used in a clinical or insurance setting to gain an understanding of likely future outcome. Further, current study findings also indicated that expected time to RTW, based on our earlier review of the literature [5], allows for the collection of RTW expectation data that is related to disability duration. As such, findings support the suggested measure as being effective for work-disability risk prediction purposes.

When conceptualizing this study we saw the potential for the relationship between RTW expectation and outcome to be influenced by who was questioning the workers about their expectations. More specifically, we saw the potential for workers to give a more “socially desirable” (and thus less accurate) response when they were asked about their RTW timeframes by an insurance representative involved in their case management. While we cannot be sure that this was not the case, we did observe that workers’ RTW estimates as recorded by their claims managers were highly predictive of compensated work-disability duration.

Our finding that some of the relationship between RTW expectations and work-disability duration was accounted for by demographic and injury variables is consistent with the idea that RTW expectations represent a self-assessed summary of claimant’s individual and contextual biopsychosocial influences [4, 30]. This finding also adds support to the idea that unpacking the reasoning behind one’s expectations, has the potential to assist in the identification of RTW facilitation opportunities, as well as obstacles that may be amenable to intervention with the aim of improving the sickness-absent worker’s RTW outcome [31].

In terms of implications for work-disability prevention, our finding that expected RTW was highly predictive of work-disability duration indicates that claims managers can use this information to gain an understanding of likely outcome. It provides a starting point for discussion of what might be done to assist workers to achieve a timely, safe and sustained RTW. If someone expects to RTW in the near future, this suggests that the injured worker has the necessary resources to return without the need for assistance or intervention (making them a “low touch” claim). However, it should be noted that this is not necessarily the case. There may be instances where expectations are unrealistic or forced, in which case there is the potential that the RTW could result in adverse effects [32]. If this is suspected, a low touch approach may not be the appropriate course of action. For persons who provide a far off estimate, this could indicate that help is needed, especially if that person’s health condition is relatively minor.

### Limitations and Methodological Considerations

When interpreting study findings the reader should be aware that the end of TTD payments does not necessarily mean RTW. There may be cases where people stopped getting TTD payments but did not RTW. This suggests that the relationship between expectations and disability duration may be stronger than we observed. While the data is consistent with the measure recommended based on our review [5], it is likely that there was inconsistency in how the question was asked. The impact of this potential inconsistency cannot be ascertained. Another limitation is that there are variables of interest (such as workplace relationships, availability of accommodations, work demands and distress) which were not available in the data. Inclusion of these variables would add further insight as to the relationship between RTW expectations and work-disability outcomes.

While previous research on this topic has often been limited by small sample sizes in single industries or focusing on specific injuries, the current findings indicate that the relationship between RTW estimates and disability duration holds true when the sample is large and varied in terms of condition and demographic characteristics. As such, the current results are expected to be generalizable to persons with a variety of conditions and socio-economic backgrounds. However, it should be noted that study data were drawn from a single WC insurance provider. As such, it is unclear if findings may be generalized to customers of other WC insurers. This is also true for persons with a work-disabling condition that is not work-related.

The current findings suggest a number of opportunities for future research. From a research perspective it raises questions concerning the malleability of RTW expectations. More specifically, can RTW expectations be changed and, if so, does this result in associated changes in work-disability outcomes? From a practical perspective, questions remain regarding whether having an understanding of RTW expectations helps claims managers in their work with sickness-absent workers’ compensation claimants. Does this information help them to identify opportunities for facilitating claimants’ RTW and remove barriers that are impeding their progress?

## Conclusion

Study findings add to the body of knowledge indicating a relationship between RTW expectations and RTW outcomes. They demonstrate a relationship that is present when type of condition is varied, and when the sickness-absent worker is questioned by an insurance representative involved in the worker’s claim management. While reported RTW expectations share explanatory power with other variables found in administrative databases, findings indicate that additional insight into likely work-disability duration can be gained through asking the workers when they expect to be back at work.

## Footnotes

1 Of the 3173 claimants excluded for missing information, 170 were missing perceived pain, 186 were missing age, 538 were missing tenure, 344 were missing comorbidity, 410 were missing prior injury, and 1251 were missing industry.
